# Modified DrySeal Sheath U-turn Technique with Sheath Escalation and a Triple Coaxial System for Complex Gore Excluder Iliac Branch Endoprosthesis Placement: A Retrospective Cohort

**DOI:** 10.1016/j.ejvsvf.2026.09.002

**Published:** 2026-09-23

**Authors:** Yuta Tajima, Yoshihisa Tamate, Ai Ishizawa, Tetsuo Watanabe

**Affiliations:** Department of Cardiovascular Surgery, Sendai City Hospital, Sendai, Japan

**Keywords:** Aorto-iliac aneurysm, Endovascular aneurysm repair, Iliac branch endoprosthesis, Internal iliac artery, Triple coaxial sheath system, U-turn DrySeal sheath technique

## Abstract

**Objective:**

Standard Gore Excluder Iliac Branch Endoprosthesis (IBE) placement precedes endovascular aortic repair (EVAR) main body deployment. This sequence is unavailable after previous EVAR and may be unsuitable with short common iliac arteries or a limited renal to internal iliac artery (IIA) distance. The original ipsilateral U-turn technique enables post main body IBE placement by redirecting a 12 F sheath towards the IIA through the same femoral access. Limited working space within the sheath and wire device interaction may impede delivery. This paper describes two modifications intended to address these delivery limitations: U-turn sheath escalation to ≥ 14 F (modification 1) and a 20/16/12 F triple coaxial sheath system (TCSS). Procedural feasibility and early outcomes for the original and modified U-turn techniques are reported.

**Methods:**

Consecutive limbs treated at one centre with the original or modified U-turn technique between December 2023 and December 2025 were reviewed retrospectively; outcomes were summarised descriptively.

**Results:**

The technique was used in 46 limbs of 34 patients: 37 during initial EVAR and nine after previous EVAR. The original 12 F technique, modification 1, and TCSS were used in 15, 18, and 13 limbs, respectively. Technical success was achieved in all limbs. Computed tomography within one month showed no type I or III endoleak; one bridging stent graft occluded in the TCSS group. There were no pelvic ischaemic complications or external iliac artery dissections or perforations. Three percutaneous closure failures required suture repair.

**Conclusion:**

The U-turn approach enabled post-main body IBE placement in selected patients. When access and U-turn space were adequate, the U-turn sheath was generally escalated to ≥ 14 F; the original 12 F configuration was retained when either was limited, and TCSS was reserved for bridging expected to benefit from pathway separation or distal support. These off label strategies require careful planning. Prospective multicentre studies should assess reproducibility, comparative safety, and durability.

## Highlights

• U-turn sheath escalation increased sheath size from 12 F to 14 F or larger.
• A 20/16/12 F triple coaxial system separated pathways and provided distal support.
• Access, U-turn space, and device working length guided configuration selection.
• Post-main body iliac branch endoprosthesis placement succeeded in all 46 limbs.
• Early imaging showed no type I or III endoleak; one bridging stent graft occluded.

## Introduction

International guidelines recommend preserving flow to at least one internal iliac artery (IIA) during endovascular aortic repair (EVAR) whenever feasible to reduce pelvic ischaemic complications.^1^^,^^2^ This is a minimum target and does not imply sacrifice of a patent contralateral IIA. The Gore Excluder Iliac Branch Endoprosthesis (IBE; W. L. Gore & Associates, Flagstaff, AZ, USA) is an established option for IIA preservation in aorto-iliac aneurysm repair.^3^, ^4^, ^5^, ^6^, ^7^, ^8^ In selected patients requiring bilateral iliac treatment, preservation of both IIAs may be considered when anatomy is suitable and the added procedural risk is acceptable.^3^^,^^6^ Standard IBE first sequencing places the IBE before the bifurcated main body and subsequent iliac limb bridging.^3^^,^^5^

The standard sequence is unavailable after previous EVAR^9^ and may also be precluded during initial EVAR by insufficient common iliac artery (CIA) length or limited renal to IIA distance.^10^, ^11^, ^12^, ^13^, ^14^, ^15^, ^16^ Additional IBE strategies include up and over approaches,^17^^,^^18^ combined upper extremity and femoral access,^19^ ipsilateral deployment,^20^ and the ipsilateral U-turn DrySeal sheath technique.^21^ Distal IIA branch landing, including superior gluteal artery landing, may extend applicability when the IIA main trunk is unsuitable but increases bridging complexity.^22^

A previous report described the original U-turn technique using a 12 F U-turn sheath and an ipsilateral through and through wire.^21^ The present study added two modifications: modification 1 escalated the U-turn sheath from 12 F to 14 or 16 F within an enlarged outer working sheath; modification 2 added a 12 F inner sheath through the 16 F U-turn sheath into the IIA, creating a 20/16/12 F triple coaxial sheath system (TCSS) that separates the through and through wire from the embolisation and device delivery pathway while providing distal support. This paper also describes four adjunctive manoeuvres, practical configuration selection, working length constraints, and device related precautions, and evaluates procedural feasibility and early outcomes in a consecutive cohort.

## Design

This retrospective, single centre cohort comprised consecutive limbs treated with the original or modified U-turn technique at Sendai City Hospital between December 2023 and December 2025. The denominator included only U-turn treated limbs; conventional IBE first and non-U-turn IIA preservation or reconstruction strategies were excluded. The study was approved by the institutional review board (approval no. 20240121; approved on 2 December 2023), and the requirement for individual informed consent was waived.

## Methods

For descriptive reporting, timing was classified per limb as same session IBE placement during initial EVAR or additional IBE placement after previous EVAR. A patient could contribute one limb to each category. Each limb was counted once in limb based analyses; each staged session was counted separately in procedure based analyses. Conventional IBE first deployment was preferred when feasible. Additional IBE strategies were considered when it was unavailable after previous EVAR or anatomically unsuitable during initial EVAR. Bilateral IIA preservation was selected individually when bilateral iliac treatment was required, both IIAs were amenable to reconstruction, and standard bilateral IBE first sequencing was unsuitable because CIA length or renal to IIA distance was limited, or unavailable after previous EVAR.

### Practical selection of the U-turn configuration

Figure 1 summarises this retrospective framework; selection remained at operator discretion without pre-defined numerical thresholds. Iliofemoral access was assessed for compatibility with the intended outer sheath according to calibre, calcification, and tortuosity. U-turn space was estimated on pre-operative computed tomography (CT) and reassessed after flared limb deployment.

**Figure 1:**
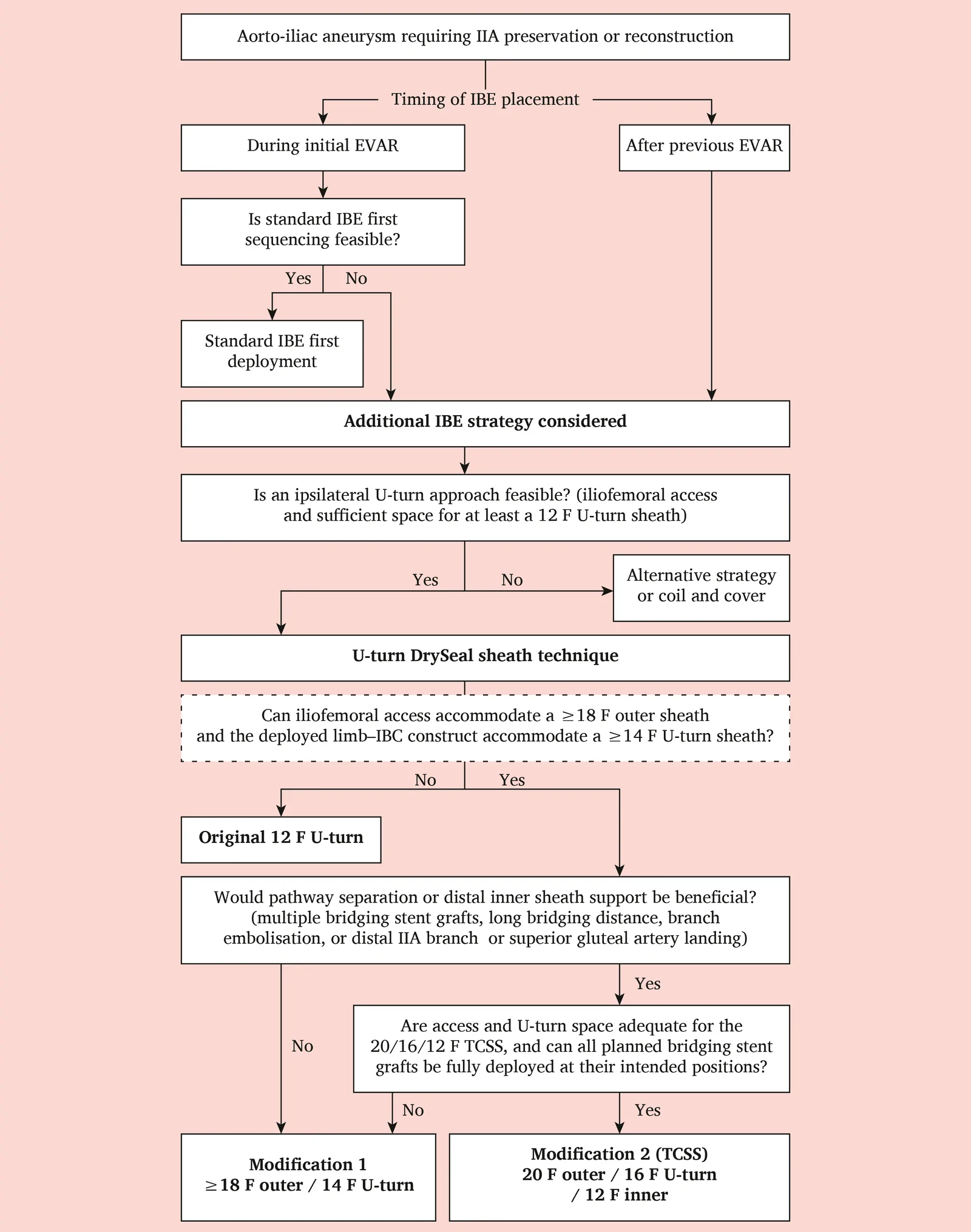
Practical framework for Gore Excluder Iliac Branch Endoprosthesis timing and U-turn configuration selection. Standard IBE first placement was preferred when feasible. After modification 1 was introduced, a ≥ 18 F outer sheath with a 14 F U-turn sheath was generally selected when access and U-turn space were adequate; the original 12 F configuration was retained when either was limited. TCSS was reserved for cases expected to benefit from pathway separation or distal inner sheath support during bridging, provided that access and U-turn space were adequate for the 20/16/12 F configuration and all planned bridging stent grafts could be fully deployed at their intended positions. This retrospective framework is not a validated algorithm. EVAR = endovascular aortic repair; IBC = iliac branch component; IBE = Gore Excluder Iliac Branch Endoprosthesis; IIA = internal iliac artery; SESG = self expanding stent graft; SGA = superior gluteal artery; TCSS = triple coaxial sheath system.

After modification 1 was introduced, a ≥ 18 F outer/14 F U-turn configuration was generally preferred when iliofemoral access and U-turn space were adequate, including IIA main trunk bridging with a single self expanding stent graft (SESG), unless pathway separation or distal inner sheath support was anticipated. The original 12 F technique was retained when access or U-turn space was limited. TCSS was reserved for cases expected to benefit from pathway separation or distal inner sheath support, including multiple SESGs, a long bridging distance, branch embolisation, or distal IIA branch or superior gluteal artery (SGA) landing, provided that access and U-turn space were adequate for the 20/16/12 F configuration and all planned bridging stent grafts could be advanced to their intended positions and fully deployed. These preferences were retrospective and unvalidated. Procedures were performed or directly supervised by operators experienced in IBE placement and familiar with the original U-turn technique.

In the original technique, a 12 F Gore DrySeal Flex Introducer Sheath (W. L. Gore & Associates, Flagstaff, AZ, USA) was turned within the deployed IBC towards the IIA over an ipsilateral through and through wire.^21^ Principal limitations were intraluminal crowding, wire–device interaction, delivery resistance, and lack of distal sheath support.

### Modification 1: U-turn sheath escalation

For modification 1 and TCSS, an 18 F or larger DrySeal sheath was shortened from 33 cm to ≤ 16 cm using a pointed scalpel and straight scissors. The entire cut circumference was inspected visually and palpated through sterile gloves for protruding reinforcement wire, delamination, or a sharp or irregular surface. Only sheaths passing this assessment were used; otherwise, re-trimming with repeat inspection or disposal was required. The shortened sheath was introduced as the outer working sheath over an extra stiff guidewire (stiff wire 1; Fig. 2A). Manual shortening was outside the applicable instructions for use. Figure 2 depicts the configuration rather than a fixed sequence, which varied with previous EVAR status and workflow.

**Figure 2:**
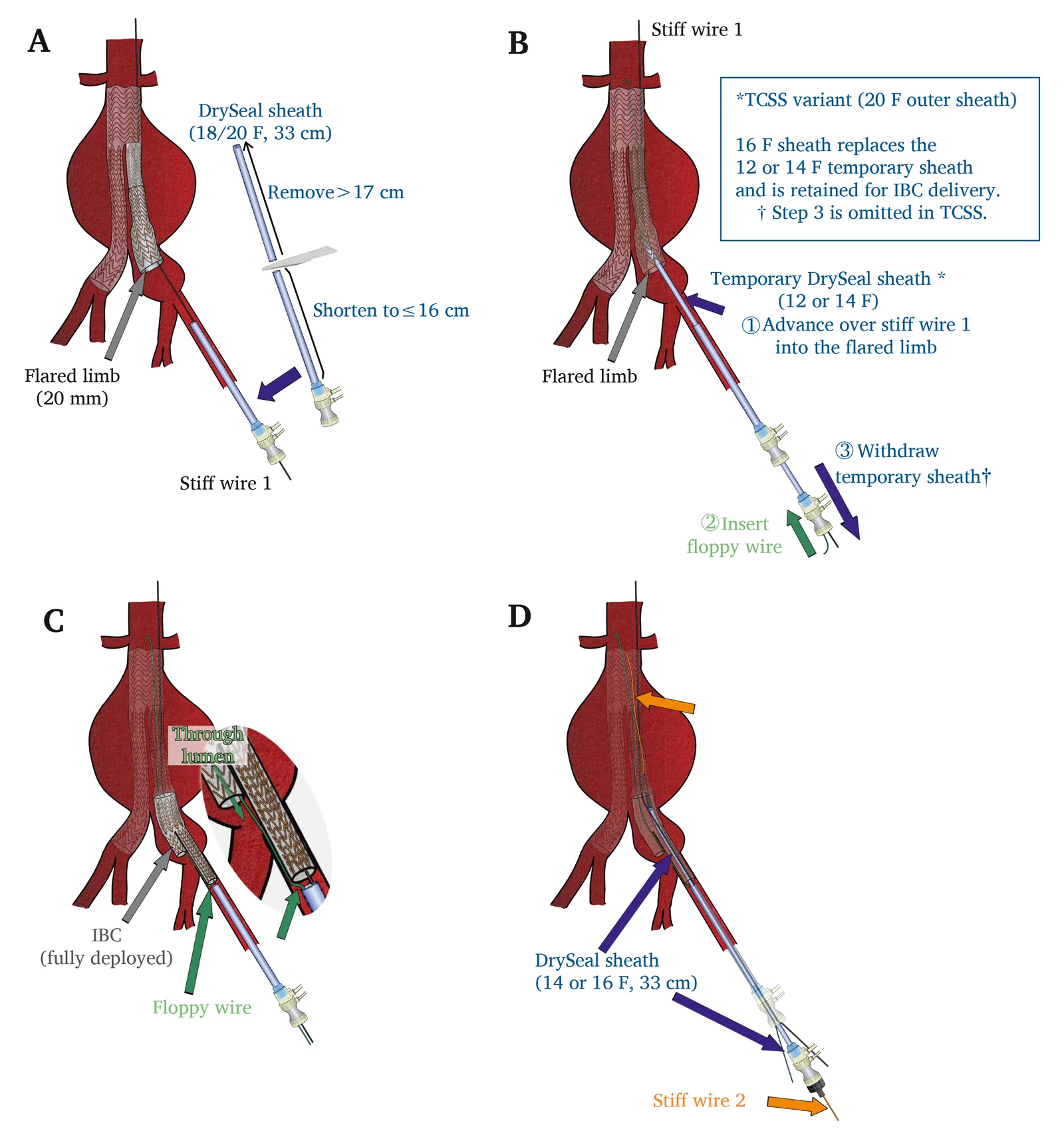
Preparation and wire management before U-turn formation. (A) An 18 F or 20 F DrySeal sheath is shortened to ≤ 16 cm and used as the outer working sheath. (B) A temporary 12 F or 14 F sheath facilitates floppy wire entry into the flared limb; in TCSS, a retained 16 F sheath can serve as the IBC delivery conduit. (C) The floppy wire is back loaded through the IBC, which is deployed within the flared 20 mm iliac limb. (D) Before through and through access, the U-turn sheath is advanced over a temporary second stiff guidewire beyond the IBC flow divider to limit traction on the IBC–flared limb junction. TCSS = triple coaxial sheath system; IBC = iliac branch component.

After outer sheath placement, a floppy wire was introduced in parallel with stiff wire 1 into the flared limb. If direct entry was difficult, a temporary 12 F or 14 F DrySeal sheath was advanced over stiff wire 1 to guide the floppy wire (tip 1; Fig. 2B), then withdrawn. In TCSS, a 16 F sheath could instead be retained as the IBC delivery conduit.

The floppy wire was back loaded through the IBC through lumen, and the IBC was advanced over stiff wire 1 and deployed at the planned axial position with the intended overlap within the 20 mm flared iliac limb (Fig. 2C). Before through and through access was established, a temporary second stiff guidewire was introduced, and the U-turn sheath was advanced over it beyond the IBC flow divider (tip 2; Fig. 2D). This avoided traction dependent advance across the flow divider after wire externalisation and was intended to limit force transmission to the IBC–flared limb junction.

The temporary second stiff wire was removed after the U-turn sheath crossed the flow divider, and the floppy wire was snared and externalised (Fig. 3A). A 12 F dilator was inserted into the U-turn sheath to reduce the turning radius (tip 3; Fig. 3B). The sheath–dilator assembly was turned towards the IIA by gentle, coordinated push–pull manoeuvres with balanced tension on both externalised wire ends. The dilator was advanced only as far as required; a 45 cm dilator was not advanced beyond the depth of a 33 cm dilator. Forceful contact with the endograft was avoided.

**Figure 3:**
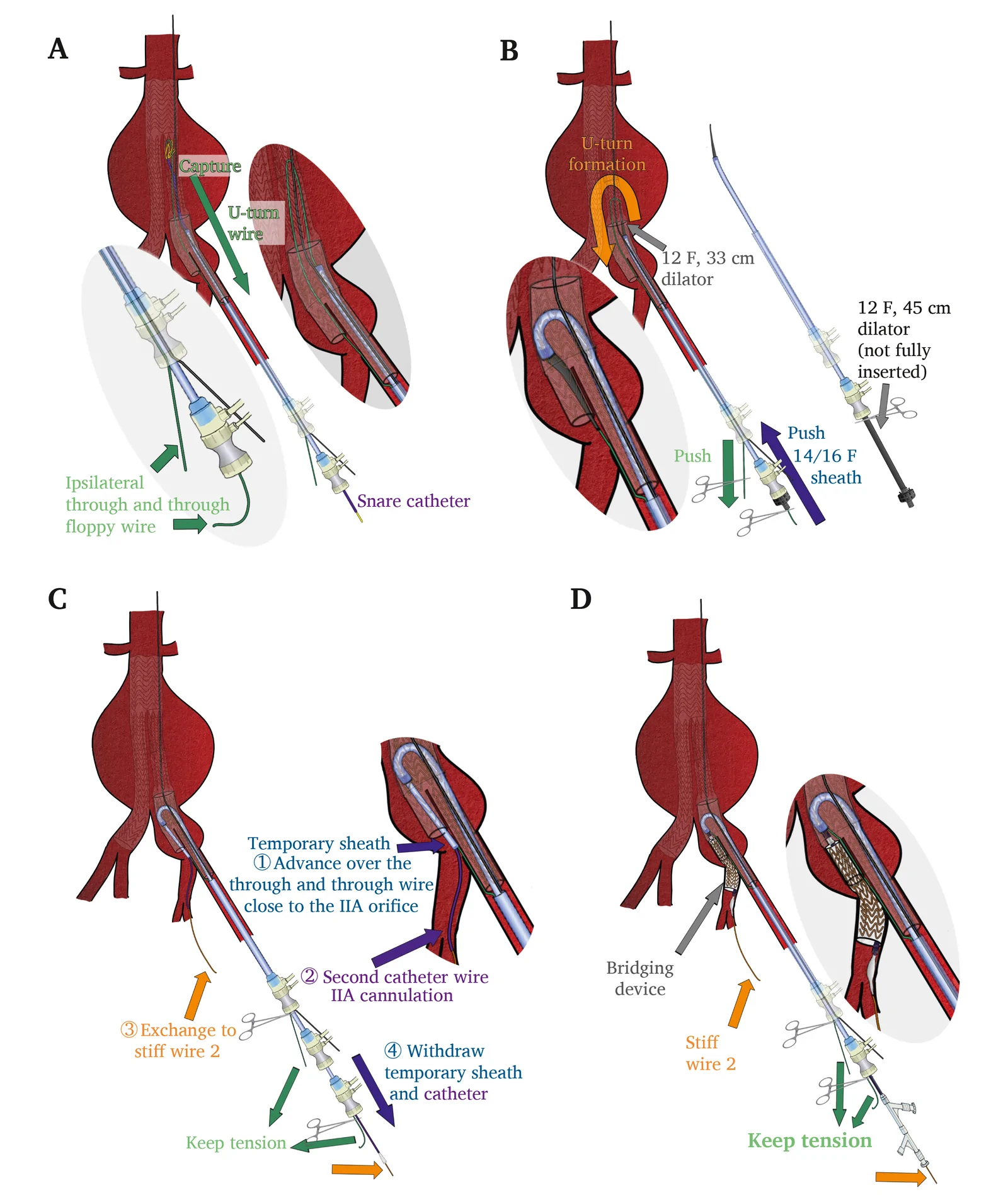
U-turn formation, adjunctive near orifice IIA cannulation, and bridging device delivery. (A) After the U-turn sheath crosses the IBC flow divider, the floppy wire is snared and externalised. (B) A 12 F dilator facilitates U-turn formation; through and through wire tension is adjusted under fluoroscopy to preserve the configuration. (C) When direct IIA cannulation is difficult, a temporary sheath near the IIA orifice supports a second catheter–wire system. (D) The bridging device is delivered over a stiff wire positioned in the IIA. IIA = internal iliac artery; IBC = iliac branch component.

During wire externalisation, U-turn formation, and device delivery, the IBC, flared limb, and adjacent modular overlap zones were monitored fluoroscopically. Manipulation was stopped and the configuration reassessed if resistance, deformation, or component movement was suspected. After U-turn formation, gentle tension was maintained on the through and through wire, and repeated axial movement of the sheath was minimised.

The target IIA or distal branch was cannulated, and the floppy wire was exchanged for stiff wire 2. If direct cannulation was difficult, a long temporary sheath was advanced near the IIA orifice over the through and through wire, and a second catheter–wire system was introduced in parallel (tip 4; Fig. 3C). The temporary system was removed after exchange to stiff wire 2. The bridging device was delivered over stiff wire 2, with through and through wire tension adjusted under fluoroscopy, and transiently increased as needed when device advance tended to straighten the U-turn sheath (Fig. 3D).

### Modification 2: Triple coaxial sheath system

TCSS comprised a 20 F outer sheath and a 16 F U-turn sheath. After U-turn formation, a 12 F, 45 cm DrySeal inner sheath was advanced over stiff wire 2 into the IIA (Fig. 4A). When required, branch embolisation was performed through a guiding sheath introduced via the inner sheath, separate from the through and through wire (Fig. 4B). Internal iliac component (IIC) delivery, including additional IICs in multiple bridging cases, was performed with distal support through the inner sheath (Fig. 4C). Device compatibility and delivery system working length were assessed during planning to confirm that each planned bridging stent graft could be advanced to its intended position and fully deployed (Fig. 4D). Key steps are shown in the Supplementary Video.

**Figure 4:**
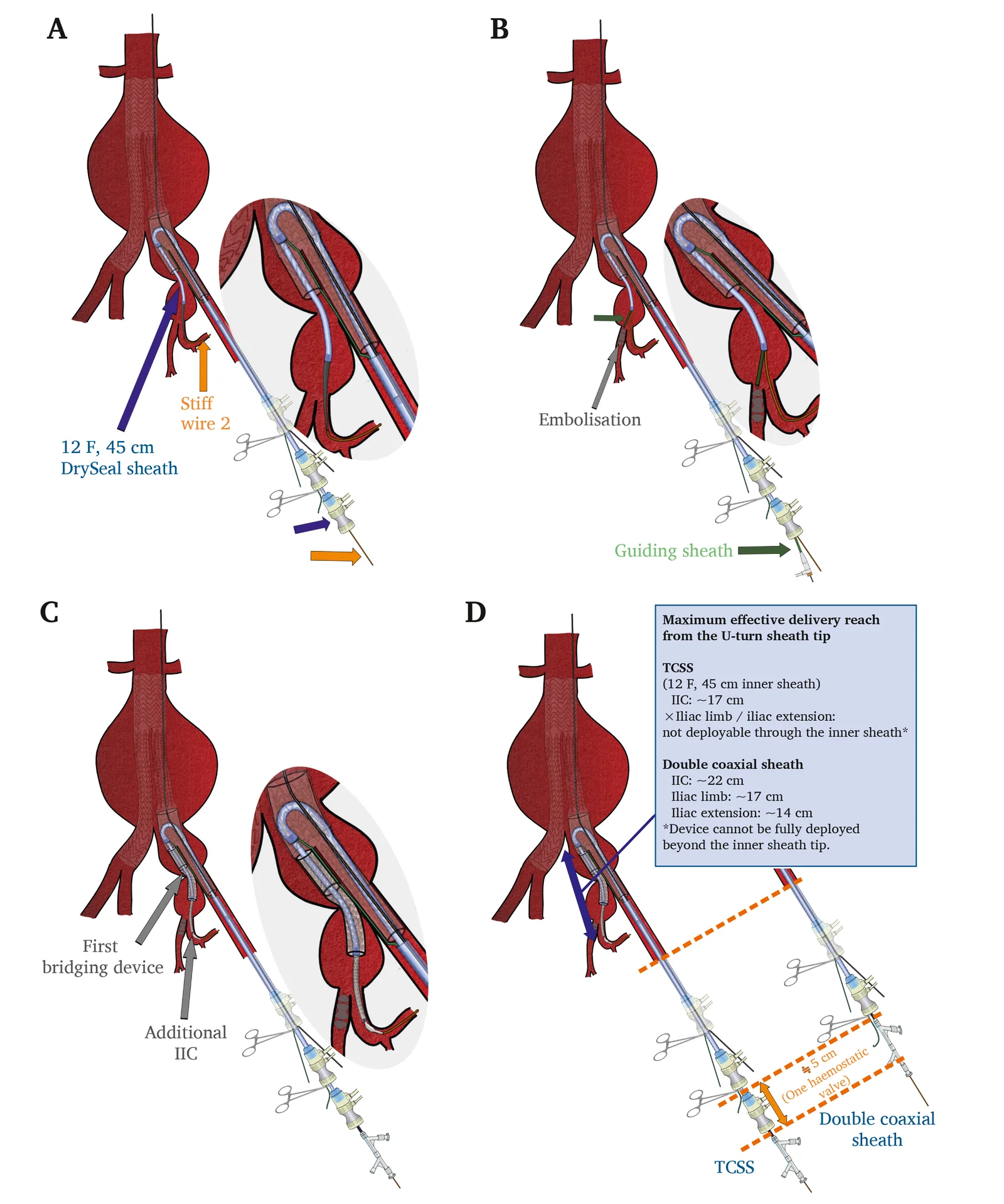
Triple coaxial sheath system and working length considerations. (A) A 12 F, 45 cm inner DrySeal sheath is advanced through the U-turned 16 F sheath into the IIA, completing the 20/16/12 F TCSS. (B) Branch embolisation is performed through a guiding sheath separate from the through and through wire. (C) The inner sheath provides distal support for IIC delivery, including in multiple bridging cases. (D) The additional haemostatic valve reduces IIC reach by approximately 5 cm compared with the double coaxial configuration. IIA = internal iliac artery; TCSS = triple coaxial sheath system; IIC = internal iliac component.

Technical success was defined as successful bridging from the IBC to the target IIA or distal branch without type I or III endoleak on completion angiography. Outcomes included planned U-turn abandonment, early type I or III endoleak, bridging stent graft occlusion, pelvic ischaemic complications, percutaneous common femoral artery closure failure, external iliac artery dissection or perforation, estimated blood loss, fluoroscopy time, and radiation dose. Early imaging outcomes were assessed on CT within one month. Data were summarised descriptively without between group statistical comparisons. Estimated blood loss, fluoroscopy time, and radiation dose were summarised per procedure because they reflected the complete workflow, including concomitant initial EVAR, embolisation, and unilateral or bilateral IBE placement.

## Results

The U-turn technique was used in 46 limbs of 34 unique patients: 37 limbs in 28 patients during initial EVAR and nine limbs in seven patients after previous EVAR. One patient underwent unilateral treatment during initial EVAR and contralateral treatment later, contributing one limb to each timing category but counted once overall. Bilateral IIA preservation was achieved in 12 patients: nine underwent simultaneous bilateral treatment during initial EVAR, two underwent staged bilateral treatment after previous EVAR, and one underwent the cross category staged treatment described above.

The original 12 F technique, modification 1, and TCSS were used in 15, 18, and 13 limbs, respectively. All modification 1 limbs used a 14 F U-turn sheath. Multiple bridging SESGs were required in 10 of 13 TCSS limbs, and SGA landing was used in five limbs (Table 1). No shortened DrySeal sheath required re-trimming or disposal; all passed final visual and tactile inspection before insertion.

**Table 1:** Technical details, early outcomes, access related events, and procedure based operative metrics of the original and modified U-turn DrySeal sheath techniques (*n* = 34).

Variables		Original technique (*n* = 15 limbs)	Modification 1∗ (*n* = 18 limbs)	Modification 2 (TCSS†) (*n* = 13 limbs)
Same session IBE during initial EVAR		11 (73)	16 (89)	10 (77)
Additional IBE after previous EVAR		4 (27)	2 (11)	3 (23)
U-turn sheath size – F		12	14	16
Number of bridging stent grafts per limb, mean		1.27	1.44	2.15
Multiple bridging stent grafts required		3 (20)	6 (33)	10 (77)
Distal landing site,	IIA main trunk	14 (93)	17 (94)	8 (62)
	IIA branch (all SGA)	1 (7)	1 (6)	5 (39)
Bridging stent graft occlusion		0 (0)	0 (0)	1 (8)
Type I or III endoleak		0 (0)	0 (0)	0 (0)
Pelvic ischaemic complications‡		0 (0)	0 (0)	0 (0)
Access related events§	Percutaneous closure failure	1 (7)	0 (0)	2 (15)
	EIA dissection or perforation	0 (0)	0 (0)	0 (0)
Procedure based operative metrics‖		Overall procedures	Original only procedures	Procedures including any modified technique
Number of procedures		37	11	26
Estimated blood loss – mL		220 (80, 325)	240 (80, 405)	139 (80, 230)
Fluoroscopy time – min		117 (66, 141)	123 (77, 189)	118 (58, 143)
Radiation dose, cumulative air kerma – mGy		668 (374, 977)	936 (502, 1104)	629 (265, 909)

Data are presented as *n* (percentage) or median (interquartile range), unless otherwise stated. Unless otherwise specified, data are presented on a limb-based basis. Percentages were calculated using the number of limbs in each technique group as the denominator.

Timing categories were assigned per treated limb. One patient contributed one limb to each category because one side was treated during initial EVAR and the contralateral side in a separate later procedure; this patient was counted once in the overall cohort.

TCSS = triple coaxial sheath system; EVAR = endovascular aneurysm repair; IBE = Gore Excluder Iliac Branch Endoprosthesis; IIA = internal iliac artery; SGA = superior gluteal artery; CFA = common femoral artery; EIA = external iliac artery; IQR = interquartile range.

∗ The Modification 1 group comprised U-turn sheath escalation without TCSS; all limbs in this group used a 14 F U-turn sheath.

† TCSS (Modification 2) built on a 16 F U-turn sheath by adding a 12 F inner sheath, creating the 20/16/12 F configuration.

‡ Pelvic ischaemic complications included treated side buttock claudication.

§ Access-related events were tabulated according to the U-turn sheath category. Technique classification was based on the U-turn sheath category and use of TCSS, not on the maximum outer sheath size used during the entire procedure. In some concomitant initial EVAR procedures, a larger outer sheath was used for procedure requirements unrelated to U-turn classification; therefore, some limbs classified as the original technique used ≥ 18 F outer sheaths, and some modification 1 limbs with a 14 F U-turn sheath used a 20 F outer sheath. Accordingly, access-related events should be interpreted descriptively by U-turn category and not as a direct comparison of outer sheath diameter. All CFA access sites were pre-closed. All percutaneous closure failures were managed by suture repair of the sheath removal site through a small skin incision.

‖ Estimated blood loss, fluoroscopy time, and radiation dose were analysed on a per-procedure basis and included the entire procedural workflow, including, when performed, concomitant initial EVAR, branch embolisation, and uni- or bilateral IBE placement. IBE only procedures, concomitant initial EVAR procedures, and simultaneous bilateral IBE procedures were each counted as one procedure. For staged treatments, each procedure was counted separately. Procedures in which at least one limb was treated using Modification 1 or Modification 2 were classified as procedures including any modified technique. These data are presented descriptively without statistical comparison.

Technical success was achieved in all 46 limbs, and no planned U-turn approach was abandoned. No component movement or deformation requiring intra-operative correction was documented. CT within one month showed no type I or III endoleak. One bridging stent graft occluded in the TCSS group. There were no pelvic ischaemic complications, including treated side buttock claudication.

Percutaneous common femoral artery closure failed in one original technique limb and two TCSS limbs; no failures occurred in modification 1 limbs. All three were managed by suture repair through a small incision. No external iliac artery dissection or perforation occurred. These findings are descriptive because technique categories reflected the U-turn configuration rather than the maximum outer sheath used during the procedure.

Procedure based metrics were available for 37 procedures: 11 original only procedures and 26 including at least one modified technique. Median estimated blood loss was 220 mL overall, 240 mL in original only procedures, and 139 mL in procedures including at least one modified technique. Corresponding median fluoroscopy times were 117, 123, and 118 minutes, and cumulative air kerma values were 668, 936, and 629 mGy. Because these metrics reflected the complete workflow, they could not isolate the incremental burden of the U-turn component or either modification.

## Discussion

In this selected cohort, all 46 limbs were treated successfully without planned abandonment. The short ipsilateral route avoids aortic arch traversal and an up and over course across the main body endograft^17^^,^^18^ and permits delivery of approved SESGs.^21^ The original 12 F sheath shares a limited lumen between the through and through wire and bridging device and provides no distal IIA sheath support. Modification 1 enlarges the working lumen; TCSS additionally separates the pathways and provides distal support. TCSS was used mainly for multiple SESGs or SGA landing, consistent with this role, but its incremental benefit could not be quantified because wire–device interaction and delivery resistance were not recorded prospectively.

Standard IBE first deployment remains preferred. The U-turn route may be considered after previous EVAR or when CIA length or renal to IIA distance is insufficient for standard sequencing.^10^, ^11^, ^12^, ^13^, ^14^, ^15^, ^16^ Post-main body placement may allow limited axial adjustment of the IBC while adequate overlap and gate orientation are maintained, potentially reducing the required renal to IIA treatment length and dependence on exact bridge length matching.^17^^,^^18^^,^^23^ Here, the U-turn formation segment refers to the approximately 3 cm portion of the iliac limb in which the IBC main trunk is intended to be deployed.^3^ More proximal IBC placement may shorten the required treatment length but place more of this segment proximal to the fully expanded flare, reducing the space available for U-turn formation.

Pre-operative CT was used to predict the position of this segment and whether the iliac limb would expand adequately throughout it; final judgement followed flared limb deployment. A well expanded 20 mm flare encompassing most of the segment provided sufficient space for the 16 F U-turn sheath used in TCSS; this was a clearly feasible condition, not a minimum requirement. A 14 F U-turn sheath could also be formed when part of the segment extended into the proximal 16 mm portion of the iliac limb. No numerical cutoff could be derived; the practical requirement was sufficient space for a smooth U-turn throughout the segment.

Larger U-turn sheaths require larger bore access and more turning space. Small calibre, calcified, stenotic, or tortuous access, or incomplete expansion of the U-turn segment may favour the original 12 F configuration. After modification 1 was introduced, an ≥18 F outer/14 F U-turn configuration was generally used when access and U-turn space were adequate. TCSS was used selectively when pathway separation or distal inner sheath support was anticipated, access and U-turn space were adequate for the 20/16/12 F configuration, and all planned bridging stent grafts could be fully deployed at their intended positions (Figure 1, Figure 4 and Fig. 4).^24^ These preferences are unvalidated. Distal branch landing may extend applicability but increases complexity.^22^ Bridging stent grafts should be selected according to anatomy, device behaviour, approved indications, and availability.^25^^,^^26^

Bilateral IIA preservation was selected, not routine, to avoid intentional sacrifice of a patent, reconstructable IIA during bilateral iliac treatment; this potential benefit must be weighed against added procedure and device related risks. Bilateral IBE placement is described in the instructions for use, but the post-main body U-turn sequence and sheath modifications used here were outside the applicable instructions for use.^3^

Manual sheath shortening removes the factory formed tapered tip and distal radiopaque marker and creates a non-factory edge in a wire reinforced shaft.^27^ Protruding wire, delamination, or edge irregularity may increase friction and cause intimal injury, dissection, perforation or rupture, and bleeding. Circumferential inspection, stiff wire guidance, fluoroscopic monitoring, and reassessment whenever resistance is encountered are essential. No external iliac artery injury occurred, but this series does not establish safety.

The manoeuvre occurs within a newly deployed IBC–flared limb construct before tissue incorporation. Because the U-turn sheath crosses the IBC flow divider and is turned over a through and through wire, traction may cause IBC migration or loss of IBC–flared limb overlap; force may also be transmitted to the flared limb–main body junction, and direct sheath or dilator contact may damage the graft.^3^ Precautions included crossing the flow divider over a second stiff guidewire before wire externalisation, limiting dilator insertion, adjusting wire tension, monitoring component positions fluoroscopically, and minimising repeated axial movement. No component movement or deformation requiring intra-operative correction was documented, and no early type I or III endoleak was detected. Nevertheless, subtle graft injury or delayed migration could not be excluded.

Early outcomes support feasibility rather than comparative safety. There were no pelvic ischaemic complications; one bridging stent graft occluded in the TCSS group, and three percutaneous closure failures required suture repair. Downsizing, up and over or upper extremity access, another post-main body IBE strategy, or coil and cover should remain available as bailout options.

This study was limited by its retrospective, single centre design, and U-turn only denominator, which introduced selection bias. Selection criteria, U-turn space assessment, and procedure difficulty were not prospectively standardised or quantified; there was no pre-specified comparator, and operator selected groups were not designed for comparison. Thus, superiority, comparative safety, and the incremental benefit or risk of bilateral preservation could not be established. Standardised patient reported outcomes were not collected. The 100% technical success rate may reflect operator experience, case selection, and centre specific planning; the learning curve and external reproducibility remain unknown. Procedure based metrics captured the entire workflow rather than the U-turn component and follow up was early. These findings therefore represent selected cohort feasibility rather than broadly generalisable evidence. Prospective multicentre studies with pre-defined criteria, operators of varying experience, standardised clinical, imaging, and patient reported outcomes, and longer follow up are needed. Bench testing could further define how U-turn space and 12 F, 14 F, or 16 F sheath size affect turning radius, kinking, and delivery resistance.

### Conclusions

In this selected cohort, technical success in all treated limbs supported the feasibility of post main body IBE placement using the original or modified U-turn configurations. U-turn sheath escalation enlarges the working lumen, whereas TCSS provides pathway separation and distal sheath support for complex IIA bridging. Standard IBE first placement remains preferred, and these off label strategies should be considered selectively when standard IBE first placement is not feasible, the access and U-turn space are adequate, and all planned bridging devices can be fully deployed with the selected sheath configuration. Prospective multicentre comparative studies with longer follow up are needed to assess reproducibility, comparative safety, and durability.

Supplementary video related to this article can be found at https://doi.org/10.1016/j.ejvsvf.2026.09.002

The following is/are the supplementary data related to this article:

Supplementary Video Modified U-turn DrySeal sheath technique for complex additional IBE placement. The video demonstrates sheath shortening and cut edge inspection, floppy wire entry, IBC deployment, U-turn sheath advance over a temporary second stiff guidewire before through and through access, U-turn formation, TCSS establishment, branch embolisation, IIC delivery with distal support, and adjunctive near orifice IIA cannulation. Narration used a computer simulated text to speech voice from an author prepared script. IBE = Gore Excluder Iliac Branch Endoprosthesis; IBC = iliac branch component; TCSS = triple coaxial sheath system; IIC = internal iliac component; IIA = internal iliac artery.

## Funding

This research did not receive any specific grant from funding agencies in the public, commercial, or not for profit sectors.

## Data availability

The data underlying this article are available from the corresponding author upon reasonable request, subject to institutional and privacy restrictions.

## Declaration of generative ai and ai assisted technologies in the writing process

During the preparation of this work, the authors used ChatGPT (OpenAI, San Francisco, CA, USA) to assist with spelling checks and English language editing. After using this tool, the authors reviewed and edited the content as needed and take full responsibility for the content of the manuscript. The narration in the Supplementary Video was generated using a computer simulated text to speech voice based on an author prepared script. All figures and tables were created by the authors.

## Conflict of interest

None.
